# Experienced Family Caregiver Perspectives on Advance Care Planning for African Americans Living With Dementia

**DOI:** 10.1093/geroni/igab046.922

**Published:** 2021-12-17

**Authors:** Karen Moss, Kathy Wright, Laurel Myers Hurst, Abigail Grieff, Karen Rose, Todd Monroe, Celia Willis

**Affiliations:** 1 The Ohio State University, Columbus, Ohio, United States; 2 Ohio State University, Columbus, Ohio, United States

## Abstract

Most existing advance care planning (ACP) programs do not meet the needs of lower socioeconomic status (SES) African American (AA) older adults living with dementia. The perspectives of experienced family caregivers are integral to achieving appropriate ACP tailoring. The purpose of this study is to describe experienced family caregiver perceptions about needs and preferences for tailoring ACP for family caregivers of lower SES AA older adults living with dementia. This qualitative, descriptive, cross-sectional study is embedded within a larger community-based participatory study aimed at intervention development. Caregivers are completing up to two interviews. Preliminary data describes themes involving Caregiver Stress and suggestions for Service Improvements addressing grief and loss pre- and post-death. Caregiver findings and other stakeholder data from healthcare providers and community leaders will guide the design of a “new normal” enhanced, preference-consistent ACP intervention to improve end-of-life care during a global pandemic that is amplifying pre-existing healthcare disparities.

